# Tutorial options for clinicians undertaking postgraduate degrees online

**DOI:** 10.15694/mep.2020.000013.1

**Published:** 2020-01-15

**Authors:** Naomi Noguchi, Fiona Fern Stanaway

**Affiliations:** 1 University of Sydney

**Keywords:** Online education, clinicians, small-group teaching, asynchronous online discussion, intensive workshop, blended mode.

## Abstract

This article was migrated. The article was marked as recommended.

**Purpose:** To determine clinicians’ preference for types of tutorial participation and their subsequent tutorial participation and performance.

**Methods:** Part-time online students undertaking an introductory postgraduate unit of study in clinical epidemiology were offered different tutorial options across semesters.

**Results:** Ungraded asynchronous online discussions were poorly attended with only 118/186 (63%) posting at least once across 10 tutorials. Allocating a 1% participation mark each week and allowing students to complete tutorial tasks individually increased participation with 148/190 (78%) completing at least 8 out of 10 tutorials. Moreover, the final assignment mark distribution lost the tail of poorly performing students. In semesters when two tutorial options were offered, 192/200 (96%) chose to work alone over asynchronous online discussion and 162/190 (85%) chose to work alone over intensive face-to-face workshops. Even when students self-selected to join graded asynchronous online discussion, only 2/8 (25%) completed at least 8 out of 10 tutorials. In students who selected to attend intensive workshops, both participation and final assignment grade were better than that observed in other students.

**Conclusions:** The majority of clinicians studying online chose to work individually. Allowing students to work alone and awarding participation marks appeared to improve both participation and knowledge attainment.

## Introduction

Online education provides greater access to education for clinicians who frequently work long and irregular hours. However, it is a challenge to engage these students who are often time-poor, particularly in online discussion forums ( Kear, 2004).

One key area that differs between face-to-face and online courses in tertiary institutions is the provision of tutorials. Traditional face-to-face tutorials are relatively informal discussions in small groups where students revise and put into practice key concepts that are being taught in the particular unit of study. In online education, such small-group teaching is often substituted by either synchronous online discussions (fixed-time video conferencing, teleconferencing) or asynchronous online discussions (online discussion forum). Blended learning courses that include a component of intensive face-to-face teaching on campus are another alternative to incorporate face-to-face tutorial discussions in online teaching.

As well as being a place to practice key skills and interact with teaching staff, tutorials can be a valuable opportunity for engaging with other students. In online education, peer-to-peer interaction is considered to play a key role in the learning experience because it enables deeper learning through dialogue, as well as enhancing motivation and reducing feelings of isolation ( Ryle and Cumming, 2007).

Of all the online substitutes for small-group teaching mentioned above, asynchronous online discussion forums are often used because they are the most flexible in terms of time and place. Currently, all of the 72 postgraduate online units of study offered by the School of Public Health at the University of Sydney use asynchronous online discussions either for graded or ungraded study activities.

Despite their frequency of use, many teachers find it challenging to encourage participation in non-mandatory asynchronous online discussions ( Kear, 2004). The main reason seems to be that asynchronous online discussions are very time-consuming for students as they need to keep up with a large number of postings, some of which are of only questionable importance ( Kear, 2004). Furthermore, many researchers have observed that low to medium level interactions tend to dominate discussions and high-level interactions are only rarely observed in asynchronous online discussions ( Zhou, 2015).

In this paper, we describe a series of discrete choice experiments where different tutorial options were provided to students. We compared student participation in tutorials across semesters when tutorial methods were modified as well as the proportion of students selecting each tutorial option within a semester when different tutorial options were offered. Tutorial options included asynchronous online discussions, intensive face-to-face workshops, and completing tutorial tasks alone without peer discussion. The aim of our study is to identify tutorial approaches that clinicians studying online prefer, and that are associated with increased student participation and either equivalent or improved performance in the final assignment. Our hypotheses were that 1. Clinicians undertaking postgraduate degrees part-time and online would prefer working by themselves; 2. Awarding small marks would increase student participation; 3. A self-selected group of students would have meaningful asynchronous online discussions; and 4. A minority of students would choose to attend face-to-face intensive workshops and find this experience beneficial. The results of our study will inform the postgraduate clinical education community of the impact of various options for delivering tutorials online.

## Methods

### Participants

All online students who are enrolled in a postgraduate unit of study, CEPI5100 Introduction to Clinical Epidemiology, at the University of Sydney in Semesters 1 and 2 2015, Semester 1 2016, Semester 1 2017 and Semester 1 2018 were included in the analyses. Semester 2 2016 and Semester 2 2017 were excluded because there was no discrete choice experiment in these semesters. This course is compulsory in a number of postgraduate coursework degrees in Medicine and Surgery at the University and the majority of the students are medical doctors in the first few years of their training.

### Contents of the course

Introduction to Clinical Epidemiology consists of 10 modules. In each module, all students watch a series of short videos explaining key concepts and complete interactive online activities before attempting a multiple-choice quiz and a set of tutorial questions that are aimed at applying skills that students learn each week. A similar set of tutorial questions was used across all semesters. The content of the course includes how to formulate clinical questions, literature searching, research study designs, critical appraisal of systematic reviews, RCTs, and harm, prognosis, and diagnostic test accuracy studies, individualising therapy and evidence-based use of health resources.

### Tutorial options

Table 1 summarises the tutorial options that were offered each semester. In Semester 1 2015, the only option was to join ungraded asynchronous online discussions each week. In Semester 2 2015, in addition to online discussions, an option to attend two seven-hour ungraded face-to-face intensive workshops was offered. In this semester, the delivery of the online discussions was also modified by increasing the class size; allocating specific questions to each student; and limiting access to answers to only those who visited the discussion website. In Semester 1 2016, we allowed students to work individually and awarded participation marks for submitting answers to the tutorial questions online by the deadline. In Semester 2 2016, the only tutorial option for online students was graded individual answer submission. In Semester 1 2017, in addition to individual answer submission, we offered options to join graded asynchronous online discussions each week or to attend weekly graded face-to-face tutorials in the evening. In Semester 2 2017, only individual answer submission was continued. In Semester 1 2018, in addition to individual answer submission, we offered an option to attend two seven-hour graded face-to-face intensive workshops on weekends and strongly recommended this option to students on the learning management site and by email.

**Table 1.** Summary of tutorial options offered from 2015 to 2018

	S1 2015	S2 2015	S1 2016	S2 2016	S1 2017	S2 2017	S1 2018
Method(s)	Asynchronous online discussion	Asynchronous online discussion OR intensive workshops	Individual answer submission	Individual answer submission (no experiment)	Individual answer submission ORasynchronous online discussion OR weekly evening tutorials	Individual answer submission (no experiment)	Individual answer submission OR intensive workshops
Participation mark	No		1% of final mark each week				
Restricted release of answers	Available to all on LMS	Posted ondiscussion forum	Made available on LMS upon submission		Available to all on LMS		
Group size for asynchronous online discussion	20-21	37-38	NA	NA	NA	NA	NA
Allocation of questions for asynchronous online discussion	No	Yes	NA	NA	NA	NA	NA

*No discrete choice experiment in these semesters.

In each semester when multiple tutorial options were offered, online students were automatically allocated to asynchronous online discussions in Semester 2 2015, and to individual online submission in Semester 1 2017 and Semester 1 2018 unless they emailed the unit coordinators at the start of the semester about their desire to undertake a different tutorial option.

When participation marks were awarded, satisfactory participation required to receive the 1% mark each week was defined as follows. For individual answer submission, students needed to make a reasonable attempt at all questions and submit their answers by the weekly due dates. For the two seven-hour intensive workshops or evening face-to-face tutorials, students needed to turn up prepared and actively participate in discussions. If students chose to join online discussions throughout each week, they were not required to attempt all questions: 0.5% was awarded for answering an allocated question, and an additional 0.5% was awarded for responding at least once to a post by another student or the tutor. Correctness of the answers was not a criterion for awarding participation marks for any tutorial option in any semester.

### Outcomes

Students’ preference for tutorial options, participation in the tutorials and knowledge attainment were assessed as outcomes. In semesters when more than one option was available, the proportion of students that selected each tutorial option was determined. For semesters when participation marks were not awarded, participation was measured by access to the online discussion board and median number of posts across all modules. For the semesters when participation marks were awarded, participation was measured by the number of tutorials for which full marks for satisfactory participation were given. Knowledge attainment was measured by the final assignment mark. In semesters when multiple tutorial options were offered, median assignment marks were compared between the different tutorial options. Two-sided p values were calculated using the Wilcoxon rank-sum test and the analyses were done using SAS 9.4 software (SAS Institute, Cary, NC, USA).

### Ethics and consent

The use of this routinely collected data for research purposes has been approved by the University of Sydney’s Human Research Ethics Committee. Participant consent was waivered for data between Semester 1 2015 and Semester 2 2017 and opt-out consent was adopted for Semester 2 2018 when the ethics approval was granted (no student opted out).

## Results/Analysis

Table 2 summarises students’ preferences for tutorial options, their subsequent participation in the tutorial option of their choice and their performance in the final assignment. In each semester included in the analyses, 150 to 200 students were enrolled in online mode. A total of 40% to 49% were female and 85% to 96% were medical doctors across all semesters.

**Table 2.** Students’ choice of tutorial option and their subsequent participation and performance

	Semester 1 2015	Semester 2 2015		Semester 1 2016	Semester 1 2017 †		Semester 1 2018	
	Ungraded asynchronous online discussion	Ungraded asynchronous online discussion (modified delivery *)	Ungraded intensive workshops	Graded individual answer submission	Graded individual answer submission	Graded asynchronous online discussion	Graded individual answer submission	Graded intensive workshops
Number	186(100%)	133(89%)	17(11%)	190(100%)	192(96%)	8(4%)	162(85%)	28(15%)
Accessed external discussion websiteat least once	160(86%)	113(85%)	NA	NA	NA	NA	NA	NA
Median posts(Q1, Q3)	2(0, 12)	1(0, 8)	NA	NA	NA	18(11,25)	NA	NA
Posted at least 1 post	118(63%)	72(53%)	NA	NA	NA	8(100%)	NA	NA
Completed at least1/10 tutorial	NA	NA	NA	187(98%)	189(98%)	8(100%)	134(83%)	NA
Completed at least5/10 tutorials	NA	NA	Dataunavailable	176(93%)	179(93%)	7(87%)	129(80%)	28(100%)
Completed at least8/10 tutorials	NA	NA	NA	148(78%)	158(82%)	2(25%)	107(66%)	NA
Completed at least10/10 tutorials	NA	NA	Dataunavailable	86(45%)	102(52%)	1(13%)	65(40%)	25(89%)
% Median final assignment marks(Q1, Q3)	69.6(60.5, 78.0)	65.7(54.3, 77.5)	64.8(50.0, 80.4)	74.1(64.0, 81.7)	71.7(61.9, 77.5)	69.1(62.5, 77.3)	76.7(66.7, 83.3)	84.2(75.4, 90.0)

There was no experiment in Semester 2 2016 or Semester 2 2017.

* The tutorial group size was increased, specific questions were allocated to each student, and access to the model answers was restricted to those who visited the discussion website.

† Only one student selected weekly evening face-to-face tutorials and as a result these were cancelled.

In Semester 1 2015, participation in the non-mandatory asynchronous discussion was very poor. Fourteen percent (26/186) did not log on to the discussion board site a single time and only 63% (118/186) posted at least one post with a median of two posts over the 10 tutorials.

In Semester 2 2015, most students selected ungraded asynchronous online discussions (133/150, 89%) and only 11% (17/150) opted to attend the ungraded intensive workshops. In the online discussion group, students’ participation did not increase although the delivery was modified. Participation data were not gathered for the ungraded intensive workshops. The median (Q1, Q3) final assignment marks were not different between groups (p=0.45, 65.7% (54.3%, 77.5%) in the online discussion group and 64.8% (50.0%, 80.4%) in the intensive workshop group).

**Figure 1.**
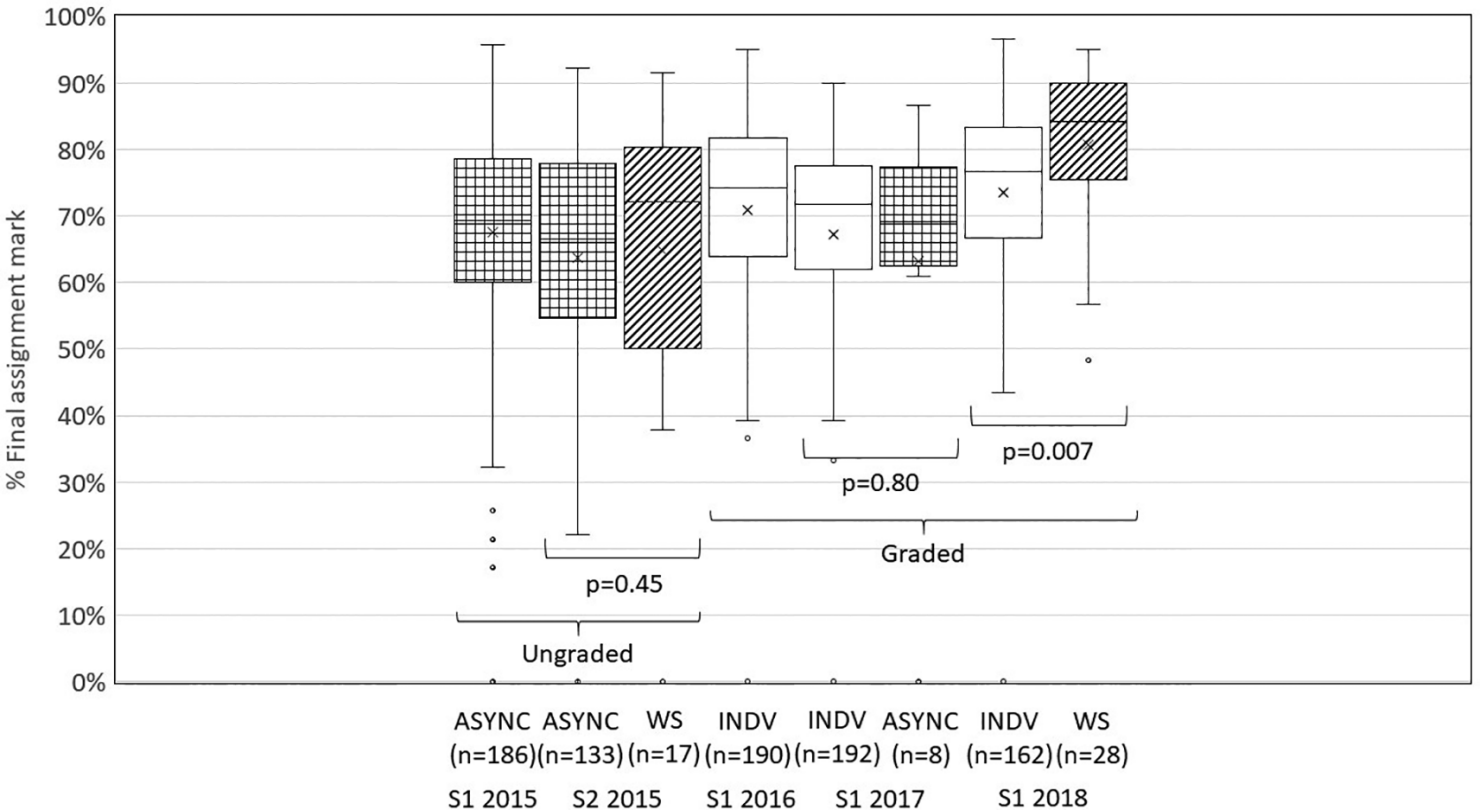
Distribution of final assignment marks by semester and tutorial option. ASYNC, asynchronous online discussion; WS, intensive face-to-face workshops; INDV, individual answer submission.

In Semester 1 2016 when all students submitted their own answers individually and small participation marks were awarded, participation increased dramatically with 98% (187/190) submitting satisfactory work for at least 1 out of 10 weekly tutorials. Seventy eight percent (148/190) submitted satisfactory work for at least 8 out of 10 tutorials. In addition, the mark distribution lost the tail of poorly performing students compared to the previous semesters ( Figure 1).

In Semester 1 2017, most students (96%, 192/200) selected graded individual answer submission and very few (4%, 8/200) chose graded asynchronous online discussions. The evening tutorial was cancelled as only one student selected this option. Even in this self-selected online discussion group, participation was not optimal with substantially fewer students in the online discussion group completing satisfactory work for at least 8 out of 10 tutorials compared to the individual answer submission group (25%, 2/8 in the online discussion group vs 82%, 158/192 in the individual answer submission group). However, 100% (8/8) in the graded online discussion group posted at least once and the median (Q1, Q3) number of postings was 18 (11, 25), which was substantially better than the first two semesters when participation marks were not offered. Seven out of the 8 students who elected to join the online discussions answered their allocated questions every week to receive 0.5% mark but 5 of them received the full mark for also responding to others’ posts only for 0 to 5 weeks. Most students posted all the comments in one module on the same day and did not return to post again. The median (Q1, Q3) final assignment marks were not different between groups (p=0.80, 71.7% (61.9%, 77.5%) in the individual submission group and 69.1% (62.5%, 77.3%) in the online discussion group).

In Semester 1 2018, most students (162/190, 85%) selected graded individual answer submission and 15% (28/190) opted to attend the graded intensive workshops. Participation rates differed somewhat by location of the students: 23% (18/77) of students living in the Sydney metropolitan area and 9% (10/111) of students living outside of the Sydney metropolitan area chose to attend the workshops. Participation in the intensive workshop group was even better than that in the individual answer submission group (89%, 25/28 attended both workshops which is equivalent to all 10 tutorials whilst only 40%, 65/162 submitted satisfactory work for all 10 tutorials online). The median final assignment mark (Q1, Q3) was also higher in the intensive workshop group (84.2% (75.4%, 90.0%) compared to the individual submission group (76.7% (66.7%, 83.3%)) (p=0.007). In the anonymous feedback survey completed by students attending the intensive workshops, 29 out of 30 indicated that they were highly satisfied with the experience. In addition to general comments that they prefer to have some face-to-face component in their learning, the following four themes emerged: ease of asking questions in person (“Face to face tute easier to ask questions”), immediate feedback (“Questions answered on the spot”); social interaction (“Interaction with everyone and being able to talk about other things”); and flexibility of not having to meet the weekly due dates (“Due to time constraints with work (often working weekends), it is difficult to meet the weekly deadlines for submission. Easier to do it all in one go.”)

## Discussion

### Online students’ preferences for tutorials

We conducted a series of discrete choice experiments for tutorial options in a group of clinicians undertaking postgraduate degrees part-time and online aimed at improving participation in tutorials and knowledge attainment. We found that the vast majority of our students chose to work individually and submit their own answers over other options. This is consistent with a previous study in an adult learning program in a university that reported that the majority of students preferred to work alone rather than in groups and identified their learning style as not collaborative ( Dixon, Dixon and Siragusa, 2007). The demographic characteristics of the students in the study by Dixon were similar to ours in that they were mature age professional workers many of whom had parenting and partnership responsibilities. We also found that despite the lack of interaction with other students, most of our students attained a high level of knowledge as reflected by their marks in the final assignment.

Although we strongly encouraged attending face-to-face tutorials, our students’ availability for and willingness to attend weekend intensive face-to-face workshops or weekly evening tutorials was limited. It could be that full-time practicing clinicians who work irregular hours and are time-poor prefer to complete learning tasks by themselves at their own pace. Although location is another important consideration, as many as 9% of students living outside of the Sydney metropolitan area were willing to travel to attend the intensive workshops. In addition, the students who attended these sessions reported being highly satisfied with their experience. In summary, working alone seems to be the most feasible and preferred option for most clinicians who are juggling work, life and studies, but face-to-face intensive workshops appear valuable to those who are willing and able to attend.

### Student participation in the tutorial option of their choice

As was found in previous studies, it can be challenging to engage part-time online students in non-mandatory asynchronous online discussions ( Kear, 2004). Participation in asynchronous online discussions in our unit of study remained low despite increasing class size so more points for discussion would be raised ( Caspi, Gorsky and Chajut, 2003), allocating specific questions to each student following the principle of team work suggest by West ( West, 2012), and restricting access to the model answers to those who visited the discussion website as an incentive to participate ( Andresen, 2009).

Participation increased dramatically when small participation marks were awarded and students were allowed to work on their own. One potential explanation of this finding could be that asynchronous online discussions are time-consuming and students do not find the gain in knowledge worth the time invested ( Kear, 2004). Our results are also supported by previous findings that awarding marks is the most influential factor for increasing student participation ( Zhou, 2015). It could be that in an increasingly competitive job market, junior doctors are undertaking master’s degrees as a way of enhancing their curriculum vitae and increasing their chances of getting onto specialist training programs. For these students, maximising marks to gain a competitive edge is an important motivation. However, to our knowledge, no previous study has described the motivations and study habits of postgraduate coursework students in Medicine.

In contrast to our original hypothesis, we found that even when students chose to join asynchronous online discussions and participation marks were given, their participation was not optimal. The majority of participants only posted their own answers to an allocated question to earn half a mark for participation. They were willing to forego the other half mark for responding to a comment from others which would require much greater effort to obtain. This type of economical behaviour has been explained by Parker *et al*. who reported that students act strategically or even illegally to invest as little effort as possible to maximise their grades ( Parker, 2003). In addition, we have observed that students appear unwilling to visit the site on multiple occasions, which is what would be required to conduct a meaningful discussion among students.

### Knowledge attainment

Increased participation when participation marks and individual answer submission was introduced was associated with better performance in the final assignment. Furthermore, final assignment marks were significantly higher in the small self-selected group of students who registered to attend intensive workshops compared to the individual answer submission group. However, it is not possible to know whether this is due to the different mode of teaching or self-selection of a group of more motivated and engaged students.

Biggs has argued that extrinsically motivated learners tend to adopt a surface approach to learning ( Biggs, 2011) and that some teachers are not comfortable motivating students by allocating marks to learning activities. In our course, however, increased participation that resulted from increased extrinsic motivation in the form of mark allocation, led to better knowledge attainment.

## Conclusion

In conclusion, when provided with different tutorial options, clinicians who study part-time and online demonstrate a strong preference for working individually rather than joining asynchronous online discussions or attending intensive face-to-face workshops. Allocating participation marks and allowing students to complete the tutorial tasks individually resulted in improved participation and knowledge attainment. Participation in asynchronous online discussions was poor even when students self-selected this option and participation marks were awarded. Face-to-face intensive workshops appear valuable for those who are able and willing to attend.

## Take Home Messages

• The majority of clinicians demonstrate a preference for working individually.
• Allowing clinicians to work alone may improve participation and knowledge attainment.
• Awarding participation marks may improve participation and knowledge attainment.
• Face-to-face workshops are valued by those who are able and willing to attend.
• Asynchronous online discussions had poor participation regardless of format.

## Notes On Contributors

**Naomi Noguchi** (ORCID: https://orcid.org/0000-0001-5662-1496) is a lecturer in clinical epidemiology at the University of Sydney. Her passion lies in teaching evidence-based practice skills to clinicians. Her main area of interest is improving online students’ participation and knowledge attainment.

**Fiona Stanaway** (ORCID: https://orcid.org/0000-0003-2104-3010) is a senior lecturer in clinical epidemiology at the University of Sydney. She has over 15 years of experience in teaching clinicians how to use evidence in their decision making. She is passionate about developing online content that is innovative and engaging for her clinician students.
