# Supplementary material for: Tutorial options for clinicians undertaking postgraduate degrees online
Source: MedEdPublish (2016). 2020 Jan 15;9:13. [Version 1] doi: 10.15694/mep.2020.000013.1 (PMC10702673; doi:10.15694/mep.2020.000013.1)
Supplement: Peer review history

# Reviewer report: Reviewer response for version 1

**DOI:** 10.21956/mep.19959.r30324

**Published:** 2020-04-30

**Authors:** Ken Masters

**Affiliations:** 1 Sultan Qaboos University

This review has been migrated. The reviewer awarded 4 stars out of 5 An interesting paper on tutorial options for clinicians undertaking postgraduate degrees online.Written and published before the COVID-19 crisis really had full effect, this paper is timely and on an important topic. The issue of engagement and participation retention has been a problem since the first days of online learning. The awarding of a small grade is an acceptable balance between carrot and stick, although the evaluation of postings is time-consuming and controversial. I think the researchers managed to get the balance right.My only major concern with the paper is the lack of detail in some areas. In their response to the first reviewer, however, I see that the authors have given some more details, and it is a pity that these were not included in Version 1 of the paper.So, while I would like to recommend the paper, I do so in the hope that the authors will create a Version 2, in which they incorporate those and other details so that the readers may gain more value from the paper.

Reviewer Expertise:

NA

No decision status is available

# Reviewer report: Reviewer response for version 1

**DOI:** 10.21956/mep.19959.r30325

**Published:** 2020-02-21

**Authors:** Barbara Jennings

**Affiliations:** 1 Norwich Medical School

This review has been migrated. The reviewer awarded 4 stars out of 5 The value of social learning is a well-established principle for evidence-based curricula. In this research article, the authors explore tutorial preferences for postgraduate students undertaking an online Introduction to Clinical Epidemiology course delivered in semesters between 2015 and 2018. Asynchronous tutorials are used on the course to facilitate tutor-student and peer-to-peer interaction.The authors describe a study in which “a series of discrete choice experiments where different tutorial options were provided”. Several cohorts were studied; with up to 192 students in an individual cohort. The methods and results sections are clear and well written – including some useful tabulated information and data.The overwhelming finding was that the clinicians in these cohorts chose to work individually. And both overall participation and learning gain (measured in assignments) were enhanced by the option to work independently & the allocation of marks specifically for participation (gaming was explored in the article). The authors explain these findings and their generalisability with reference to data from other studies in their interesting discussion. They also discuss the context for these learners – who are busy mature professionals with significant responsibilities in their wider lives.I was very interested in this well written article because I have been thinking a lot about effective and engaging technology enhanced learning (TEL) recently. I have also observed a reluctance by undergraduates and postgraduate CPD students to engage in asynchronous online discussions.The design and delivery of effective online learning for undergraduates and postgraduates is a major contemporary issue for most medical schools. Research into learning analytics and describing the evolution of curricula will be critical to the effective use of TEL. Therefore, I think this paper will be of interest to many learning technologists, course directors, and lecturers who are developing and running online modules in clinical education.I have some questions:What type of virtual learning environment was used to deliver the module? Are there any wider tips that the authors would like to share about the impact of any chosen platform for an online tutorial format?

Reviewer Expertise:

NA

No decision status is available
